# Dysregulation of miRNA in Leukemia: Exploiting miRNA Expression Profiles as Biomarkers

**DOI:** 10.3390/ijms22137156

**Published:** 2021-07-02

**Authors:** Luisa Anelli, Antonella Zagaria, Giorgina Specchia, Pellegrino Musto, Francesco Albano

**Affiliations:** 1 Department of Emergency and Organ Transplantation (D.E.T.O.), Hematology and Stem Cell Transplantation Unit, University of Bari “Aldo Moro”, 70100 Bari, Italy; luisa.anelli@uniba.it (L.A.); antonellazagaria@hotmail.com (A.Z.); pellegrino.musto@uniba.it (P.M.); 2 School of Medicine, University of Bari ‘Aldo Moro’, 70100 Bari, Italy; specchiagiorgina@gmail.com

**Keywords:** circulating miRNAs, epigenetic miRNAs, biomarkers, dysregulated expression

## Abstract

Micro RNAs (miRNAs) are a class of small non-coding RNAs that have a crucial role in cellular processes such as differentiation, proliferation, migration, and apoptosis. miRNAs may act as oncogenes or tumor suppressors; therefore, they prevent or promote tumorigenesis, and abnormal expression has been reported in many malignancies. The role of miRNA in leukemia pathogenesis is still emerging, but several studies have suggested using miRNA expression profiles as biomarkers for diagnosis, prognosis, and response to therapy in leukemia. In this review, the role of miRNAs most frequently involved in leukemia pathogenesis is discussed, focusing on the class of circulating miRNAs, consisting of cell-free RNA molecules detected in several body fluids. Circulating miRNAs could represent new potential non-invasive diagnostic and prognostic biomarkers of leukemia that are easy to isolate and characterize. The dysregulation of some miRNAs involved in both myeloid and lymphoid leukemia, such as miR-155, miR-29, let-7, and miR-15a/miR-16-1 clusters is discussed, showing their possible employment as therapeutic targets.

## 1. Introduction

Micro RNAs (miRNAs) are a subset of human non-coding RNA (ncRNA) that plays an essential role in regulating gene expression, RNA maturation, and protein synthesis [1,2]. ncRNAs have long been considered as “junk” elements; they account for about 75–90% of the human genome and are classified in two main groups according to their length: small (<200 nucleotides; miRNAs) and long (>200 nucleotides; lncRNAs) [2,3]. miRNAs are a subset of small single-stranded ncRNAs of about 19–22 nt that play a crucial role in cell growth, development, and differentiation by regulating gene expression [4,5].

Most human miRNAs map in the introns of coding genes; some may overlap with the exons, less frequently in non-coding regions or next to the 3′-UTR sites, and they are often located in the co-transcribed clusters [6,7,8]. miRNA distribution in the human genome is not random, and some chromosomes such as 1, 2, 19, and X have higher numbers of miRNAs than others [9]. Several miRNAs are located next to fragile chromosomal sites or breakpoints frequently involved in leukemia rearrangements [10,11]. miRNA synthesis starts in the nucleus with the transcription by RNA Polymerase II/III of stem-loop hairpin structures named primary miRNAs (pri-miRNAs) [12]. The endonuclease Drosha processes pri-miRNAs to produce 60–70 nt precursor miRNAs (pre-miRNAs) that are then exported to the cytoplasm and processed further by the ribonuclease Dicer to produce mature miRNA [13]. Most of the mature miRNAs bind to the “seed” region (5–8 nt long) in the 3′UTR of target mRNA molecules, induce silencing complex (miRISC) and act as post-transcriptional regulators causing mRNA degradation or translational repression via deadenylation, decapping, and exonucleolytic processes [14,15,16,17].

Moreover, other regions, such as gene promoters, 5′ UTR, or coding regions, may also be linked by miRNAs [18,19]. Up to 2000 miRNA molecules have been identified, regulating about 60% of protein-coding human genes [20,21]. One miRNA can inhibit many different mRNA transcripts, often with similar functions, and control multiple signaling pathways [22]; conversely, one mRNA transcript can be targeted by several miRNAs [23]. miRNA expression is highly tissue-specific, some miRNAs being expressed in a specific cell or tissue type; deregulation of miRNA expression has been associated with several diseases and cancers. Almost 50% of miRNAs are located near or within genes translocated in cancer [24].

By down-regulating the expression of oncogenes or tumor suppressors, miRNAs can prevent or promote tumorigenesis; therefore, they may act as oncogenes (onco-miRs) or tumor suppressors [25]. Abnormal expression of miRNA has been reported in many malignancies, in which tumor suppressors are downregulated and oncogenic miRNAs overexpressed. The roles of miRNA in leukemia pathogenesis are still emerging, and several studies have suggested miRNA expression profiles using as biomarkers for diagnosis, prognosis, and response to therapy in leukemia. These aspects will be discussed in the present review considering their mechanisms of action and the miRNAs most frequently deregulated in myeloid or lymphoid leukemias.

## 2. Epigenetic miRNAs (epimiRNAs)

MiRNA synthesis may be regulated at the transcriptional or post-transcriptional level by the same mRNA targets they inhibit [26]. The expression of some miRNAs can be silenced by DNA hypermethylation [27], whereas other miRNAs are regulated by histone modifications at their promoter regions [28]. However, some other miRNAs, defined as epigenetic-miRNAs (epi-miRNAs or epi-miRs), can directly or indirectly influence the expression of known epigenetic regulators such as DNA methyltransferases (DNMTs), HDACs, and components of PRC [29,30]. The first identified example of epi-miRNAs was the miR-29 family members (29a, 29b, and 29c); they regulate the expression of *DNMT3A* and *DNMT3B* in lung cancer and acute myeloid leukemia (AML). In vitro experiments showed that the exogenous introduction of these miRNAs in lung cancer or AML cell lines led to the reversion of the neoplastic phenotype by inhibiting different DNA methyltransferases and the consequent hypomethylation and reactivation of other target genes [31,32,33]. Subsequently, other epi-miRNAs such as miR-101, miR-140, and miR-148a/b, targeting histone-lysine N-methyltransferase EZH2, HDAC4, and DNMT3B, respectively, were identified in different cancers [34,35,36]. miR-193a is another epi-miRNA with a role in AML progression; it is silenced by the AML1/ETO chimeric protein in cases with t(8;21) translocation [37]. miR-193a is, in turn, able to repress epigenetic regulators such as HDAC3, DNMT3A, and its repressor AML1/ETO [38]. Increasing the expression of this epi-miRNA, apoptosis and differentiation are stimulated in leukemic cells due to inhibition of the AML1-ETO and other epigenetic regulators [37]. Another example of epi-miRNA is miR-217, which was found to be downregulated in chronic myeloid leukemia (CML) K562 cells that were resistant to tyrosine kinase inhibitors (TKI); it was shown that by increasing miR-217 expression in these cells, a reduction of DNMT3A and a significantly increased efficacy of TKI were detected [39]. The discovery of epi-miRNA has provided new possibilities for epigenetic-based therapeutic approaches, in which miR-29 family members, shown to be altered in different types of leukemia, are good targets.

## 3. Circulating miRNAs

**Figure 1:**
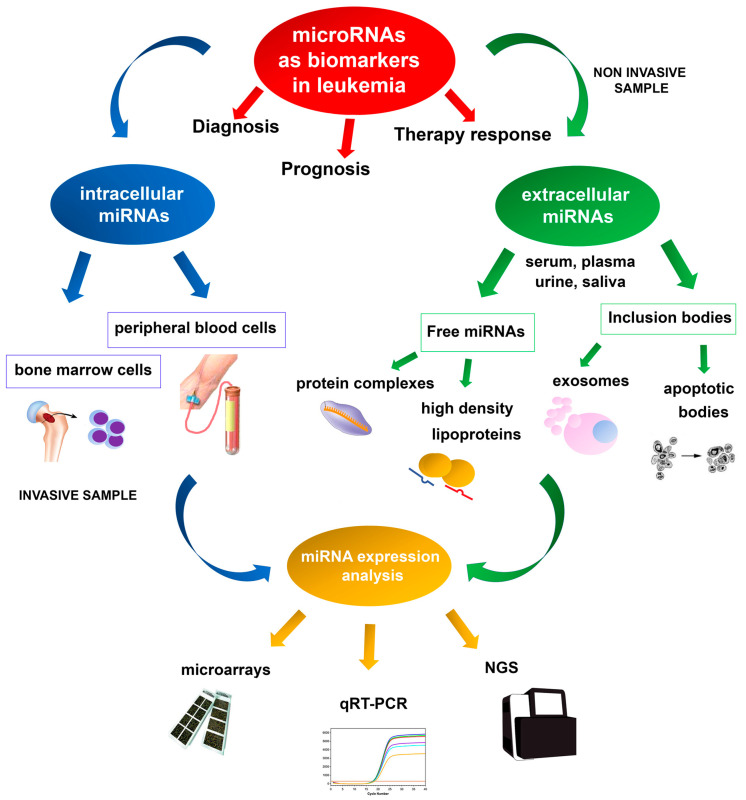
Schematic representation of miRNA employment as biomarkers in leukemia, based on invasive and non-invasive approaches for expression analysis.

Most miRNAs have an intracellular localization, but circulating miRNAs are detected in cell-free body fluids such as serum, blood, urine, and saliva (Figure 1) [40,41]. Nowadays, many researchers are investigating the possibility of exploiting microRNA dysregulated expression profiles in the bloodstream of leukemic patients as a novel liquid biopsy diagnostic tool. Circulating miRNAs are stable molecules, detectable with high sensitivity and specificity, that could be used as new potential non-invasive biomarkers of both solid and hematologic neoplasms [42,43]. Despite being cell-free RNA molecules, because of their small length circulating miRNAs are stable in different physical conditions such as wide ranges of temperature and pH [42]. Circulating miRNAs are most frequently included in protective micro-structures, named microvesicles or exosomes, secreted into body fluids [44]. Exosomes are a class of small membrane-derived vesicles, 30–140 nm in size, that are secreted by almost all cell types and contain different nucleic acids and proteins that are crucial for intracellular communication [45,46]. It has been hypothesized that the exosomes intake can cause reduced miRNA presence in cell-free body fluids in cancer patients by neoplastic cells [47]. On the other hand, increased miRNA levels in the circulation in cancer patients may be due to miRNA molecules released from neoplastic infiltrating cells and dying tumor cells. Indeed, circulating miRNAs may also be included in apoptotic bodies deriving from damaged cells [44]. Although the role of the exosomal miRNA in the pathogenesis of hematologic malignancies is not yet clear, it has been shown that exosomes derived from chronic lymphocytic leukemia (CLL) cells have a role in inducing tumor progression [48,49]. CLL cells release more exosomes in plasma than normal B-cells, and a significant overexpression of several miRNAs (miR-150, miR-155, miR-146a, and miR-29a) that promote CLL cells survival and growth has been demonstrated [48]. In particular, the expression level of miR-155 in plasma samples of CLL patients seems to be helpful as a biomarker to identify patients that may not respond satisfactorily to therapy [50]. A recent study investigated whether serum levels of miRNAs can be used as a predictive biomarker of CLL. It was found that miR-29a, miR-150-5p, and miR-155-5p were upregulated in the early stages of CLL, but that these miRNAs were poor predictive biomarkers of CLL risk [51].

Moreover, it has been shown that exosomes derived from CLL cells are actively taken by normal stromal cells, and some miRNAs such as miR-202-3p were found to be enriched in recipient cells, resulting in the downregulation of target genes [52]. Similarly, an in vitro study showed an increased level of some miRNAs (miR-146a and miR-21) in exosomes derived from multiple myeloma (MM) cell lines inducing proliferation, chemokine synthesis, and transformation of mesenchymal stem cells co-cultured with MM cells [53,54]. Moreover, an in vivo study showed that exosomes derived from MM bone marrow (BM) mesenchymal stromal cells could stimulate MM cell growth and disease progression [55]. Overall, these studies demonstrated an important role of exosomes in mediating miRNAs transfer between cancer cells and their surrounding microenvironment in B-cell malignancies.

The prognostic value and utility of circulating miRNAs as biomarkers were also demonstrated in MM. Two miRNAs, let-7b and miR-18a were associated with a poor outcome when analyzed in exosomes from treated MM patients [56]. Moreover, miR-let-7c, miR-20a, miR-103a, miR-140, and miR-185a were downregulated, whereas miR-4505 and miR-4741 were found to be higher in serum from MM patients as compared to smoldering MM cases, suggesting that exosomal miRNAs can be used as biomarkers for MM progression [57]. Furthermore, circulating miRNAs can also be used as biomarkers for drug resistance, since miR-16-5p and miR-15a-5, targeting BCL-2, were found to be significantly downregulated, whereas miR-20a-5p and miR-17-5p, targeting MYC, were upregulated in bortezomib-resistant MM patients [58].

Moreover, several circulating miRNAs in plasma or serum of AML patients could be novel, useful biomarkers, such as miR-92a, that shows a significantly lower expression in patients as compared to healthy individuals [59,60], the combination of miR-150 and miR-342, that is a potential predictor of relapse [61], or miR-181b-5p, that is significantly associated with overall survival (OS) [62]. A recent report showed that miR-155 dosage in serum-derived extracellular vesicles could be a helpful non-invasive biomarker for different hematologic malignancies such as CLL, AML, myelodysplastic syndrome, and MM [63].

However, several studies revealed different miRNA expression profiles in serum and plasma; therefore, the choice of the sample type is crucial in the use of circulating microRNAs as biomarkers [64]. Moreover, standardization of the experimental protocols to isolate exosomes and quantify circulating miRNAs is needed [65].

## 4. miRNAs and lncRNAs Interaction

Several data showed that many lncRNAs have multiple miRNA response elements (MRE), that are regions mediating reciprocal interaction; lncRNAs could therefore influence miRNA expression as they can act as sponges in both normal and cancer cells, determining several interactive networks. For example, the interaction between lncRNA *ZFAS1* and miR-150 has been demonstrated in silico and functional analyses, with *ZFAS1* inducing miR-150 downregulation. It has been demonstrated that the inhibition of *ZFAS1* in AML suppresses disease progression by inducing miR-150 overexpression and the downregulation of mRNA targets such as Myb and Sp1 [66]. Moreover, lncRNA *HOTAIRM1* targets and binds the tumor-suppressor miR-193a and modulates c-Kit expression in AML [67]. *HOTAIRM1* also showed an essential role in the pathogenesis of AML cases with the t(15;17) translocation, acting as a microRNA sponge sequestering several miRNAs [68]. Several recent papers reported different miRNA and lncRNA interactions in AML, such as *LINC01018* and miR-499a-5p, with *LINC01018* acting as a sponge of miR-499a-5p, which in turn targets *PDCD4* gene [69]. Both *LINC01018*-overexpression and miR-499a-5p knockdown suppressed AML cell proliferation and induced apoptosis, whereas miR-499a-5p transfection and silencing of *PDCD4* reversed these effects [69]. A further interesting example in AML is represented by the regulatory network of *MALAT1*/miR-146a/*CXCR4*. LncRNA *MALAT1* and *CXCR4* were upregulated, while miR-146a was downregulated in AML patients compared with healthy controls. *MALAT1* promotes migration and proliferation of AML cells by sponging miR-146a and stimulating *CXCR4* expression [70].

Moreover, lncRNA and miRNA interaction seem to be frequently involved in chemoresistance mechanisms in AML, as for lncRNA-*UCA1*/miR-125a/hexokinase 2 or *HOAX-AS2*/miR-520c-3p/*S100A4* pathways [71,72]. *UCA1* expression was found upregulated following adriamycin (ADR)-based chemotherapy, and *UCA1* knockdown enhanced the ADR cytotoxic effect in ADR-resistant AML cells. LncRNA *UCA1* directly binds and inhibits miR-125a, positively regulating its target hexokinase 2 (*HK2*) [71]. On the other hand, lncRNA *HOXA-AS2* was significantly upregulated in BM cells from AML cases after ADR-based chemotherapy, and its knockdown inhibited cell proliferation and induced apoptosis. *HOXA-AS2* and miR-520c-3p interaction were demonstrated by luciferase reporter assay, and *S100A4* was predicted as a downstream target. These data showed that both lncRNAs *UCA1* and *HOAX-AS2* may represent useful therapeutic targets for overcoming ADR-chemoresistance in AML [72].

Another interesting example is represented by the network between lncRNAs and *MYC* expression in both myeloid and lymphoid malignancies, which contributes to inhibiting apoptosis, stimulates cell proliferation, induces genomic instability and resistance to therapy [73]. Several autoregulatory loops have been reported in which *MYC* influences lncRNAs expression and is regulated by lncRNAs. In AML, lncRNA *CCAT1* interacts and inhibits miR-155, whose targets are MYC, AP-1, FOS, and c-JUN, which regulate myeloid cell differentiation [74,75]. *CCAT1* sponges miR-155 and stimulates MYC expression; interestingly, in previous studies, *CCAT1* has been reported to be activated by *MYC*, suggesting the existence of a *MYC*/*CCAT1*/miR-155 feedback loop [75]. Other identified pathways in AML are *CCAT1*/miR-490-3p/*MAPK1/MYC* and *KCNQ1OT1*/miR-326/*MYC*, in which both lncRNAs *CCAT1* and *KCNQ1OT1* sponge their target miRNAs and enhance MYC expression [75]. Other examples of lncRNA and miRNA interaction include *MEG3*/miR-147 and *UCA1*/mir-16 that have been detected in CML and are considered possible therapeutic targets in blast crisis or imatinib (IM) resistance, respectively [76,77]. Moreover, in lymphoma, the interaction between *HOTAIR* and miR-148b regulates apoptosis and the cell cycle progression of B cells. miR-148b suppresses the expression of *BMI1*, which, in turn, activates the *MAPK* and *ERK* pathways in B cells [78].

## 5. miRNAs and CircularRNAs Interaction

CircularRNAs (circRNAs) are a peculiar group of lncRNAs, composed of hundreds to thousands of nucleotides, derived from exonic, intronic (circular intronic RNAs, ciRNAs), or 5′ and 3′ UTR sequences by back-splicing of precursor mRNAs, that form a closed single-strand RNA transcript loop without a 5′ end cap and 3′ end poly(A) tail [79]. Due to their conformation, circRNAs are resistant to ribonuclease activity and are much more stable, as compared to their linear counterparts, also showing a higher sequence-conservation between species. CircRNAs have been shown to play important roles as miRNA sponges, and regulators of gene splicing and transcription, with some of them able to bind or sequester proteins, or can be translated into functional peptides [80]. As non-coding linear lncRNAs and protein-coding mRNAs, most of circRNAs have MRE regions that can bind miRNAs; therefore, all these RNA molecules compete for limited miRNAs and form a competitive endogenous RNA (ceRNA) regulatory network known as “circRNA-lncRNA-miRNA-mRNA” [81]. The majority of circRNAs are predominantly cytoplasmic and have been reported to work as ceRNAs that act as miRNA sponges. The ceRNA network plays a crucial role in physiological and pathological processes such as solid and hematologic cancers [81]. Moreover, circRNAs are widely distributed in the plasma, urine, saliva, and other human components; therefore, they can be used as promising biomarkers and therapeutic targets [82]. To date, several circRNAs have been identified as diagnostic and prognostic biomarkers in hematological malignancies, such as AML, CLL, CML, and ALL. In AML, three circRNAs have been most frequently reported as upregulated: *circDLEU2*, *circHIPK2*, and *circPAN3* involved in *circDLEU2*/miR-496/*PPKACB*, *circHIPK2*/miR-124-3p, and *circPAN3*/miR-153-5p pathways, respectively. *CircDLEU2* seems to stimulate AML pathogenesis, *circHIPK2* is involved in myeloid cell differentiation and can be considered an acute promyelocytic leukemia-associated biomarker, whereas the oncogenic circRNA *circPAN3* has a crucial role in AML drug resistance [83,84,85]. In CLL, known deregulated pathways are represented by *circCBFB*/miR-607/FZD3 inducing Wnt/b-catenin signaling, *circMTO1*/miR-337-3p/*PML* that is downregulated in CLL patients, and *circ-RPL15*/miR-146b-3p that inhibits the RAS/RAF1/MEK/ERK pathway [86,87,88]. In CML, examples of circRNA and miRNA interaction are *circTNS3*/miR-29b that stimulates leukemic cell proliferation, *circHIPK3*/miR-24 that is involved in CML progression, and *circBA9.3* that up-regulates the expression of *BCR-ABL1* and reduces TKI sensitivity [84,89]. In ALL, several reports describe the upregulation of *circPVT1* in BM samples of patients as compared to healthy individuals; it has been demonstrated that the *circPVT1* knocking down inhibits the expression of MYC and the anti-apoptotic protein BCL-2, showing that *circPVT1* could be used as a promising new therapeutic target [90].

## 6. miRNAs Expression Profile in Leukemia

MiRNA expression is tissue-specific, as different miRNAs are expressed in a specific cell or tissue type; miRNAs play a crucial role in regulating gene expression during normal hematopoiesis, acting on the self-renewal capacity of hematopoietic stem cell (HSC) and the differentiation of lineage-restricted progenitors [91,92]. Thirty-three miRNAs were identified as specifically expressed in CD34+ hematopoietic stem-progenitor cells (HSPCs) [93]. Some miRNAs such as miRNA-17, -24, 146, -155, -128, and -181, were found to be expressed in early hematopoietic cells—whereas other miRNAs, such as miRNA155, were able to control specific processes, as myelopoiesis and erythropoiesis [93], or as miR-34a and miR-17-92 clusters that have an essential role in the pro- to pre-B-cell differentiation [94]. miR-181 cluster also plays a critical role in the differentiation of hematopoietic cells as T, B, and natural killer cells or megakaryocytes [95].

Deregulation of miRNA expression has been associated with several diseases and cancers, and specific miRNA expression profiles have been reported in several hematologic neoplasms [96,97]. Microarray technologies, next-generation sequencing (NGS), and quantitative real-time PCR (qRT-PCR) are the most valuable methodologies for identifying reproducible dysregulated expression profiles in specific leukemia types (Figure 1). Unlike microarray analysis, NGS by miRNA-Seq shows high sensitivity in discovering new miRNAs and detecting whole-genome miRNA transcripts (miRNoma) with no need for previous selection. Therefore, the recent development of NGS technologies has made it possible to identify an increasing number of miRNAs involved in leukemogenesis. Several of these miRNAs may potentially be used as prognostic biomarkers, either as single miRNAs or as miRNA expression profiles.

The first evidence of the involvement of miRNAs in leukemogenesis was reported in a study of CLL aimed at characterizing a 30 Kb deletion on 13q14; the study did not identify any protein-coding genes, but a cluster of two miRNAs, miR-15a and miR-16-1, was found to be deleted or down-regulated in most of the CLL cases examined [98]. Moreover, these miRNAs have also been shown to be frequently deregulated in other solid and hematological cancer types [99,100,101], and can modulate cell cycle progression and induce apoptosis by targeting pivotal genes such as *BCL2, MCL1, CCND1,* or *WNT3A* [102,103,104].

### 6.1. Acute Myeloid Leukemia

Different studies reported specific miRNA expression profiles that distinguish between AML and acute lymphoblastic leukemia (ALL), some miRNAs being reported in different studies, such as miR-23a, miR-27a/b, miR-128a, miR-128b, miR-221, miR-222, miR-223, and let-7b [105,106]. In a study by Mi et al., four miRNAs were sufficient to distinguish between AML and ALL with an accuracy of greater than 95%, as let-7b/miR-223 being significantly upregulated and miR-128a/miR-128b downregulated in AML comparing to ALL [105]. Together, the above work showed that these identified miRNAs could be new potential markers for ALL and AML classification and diagnosis [62].

**Figure 2:**
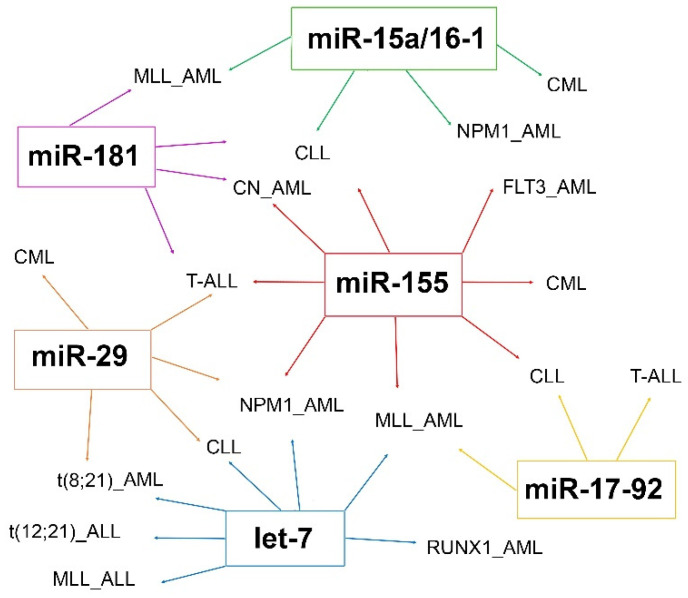
Most frequent miRNAs showing dysregulated expression in myeloid or lymphoid leukemia.

One of the miRNAs most frequently involved in AML pathogenesis is miR-155 (Figure 2), which is also commonly overexpressed in B-cell neoplasms, where it is considered an oncogenic driver of B-cell lymphoma [107]. This miRNA shows a contrasting dose-dependent function as onco-miRNA or tumor suppressor according to the expression levels. In short, a high level of expression is correlated with the antitumor effect and inhibition of AML cell proliferation. In contrast, an intermediate-low level of expression induces oncogenesis [108] and has been associated with poor prognosis in AML irrespective of specific cytogenetic or molecular aberrations [108].

Several studies have identified specific miRNA signatures in different AML subgroups defined by the classification of myeloid neoplasms [109] (Table 1), and a correlation was detected with cytogenetics alterations [110,111,112], prognosis, and clinical characteristics [65].

In AML with the t(15;17) translocation, the upregulation of miRNAs located in the 14q32 imprinted domain (miR-127, miR-154, miR-154∗, miR-299, miR-323, miR-368, and miR-370) was reported in a first study [110], whereas a set of partially overlapping strongly upregulated microRNAs (miR-382, miR-134, miR-376a, miR-127, miR-299–5p, and miR-323) was described by Jongen-Lavrencic et al. [111]. In another study, the overexpression of miR-224, miR-368, and miR-382 was detected [112].

AML with t(8;21) showed high miR-126/126∗ [112] and miR-146a expression with decreased miR-133a [110]; other evidence showed a set of down-regulated miRNAs, including two members of a known tumor suppressor microRNA family, let-7b and let-7c [111], that was previously found to be involved in other cancers [113].

In AML with inv(16), a high level of miR-99a, miR-100, and miR-224 expression or of miR-126/126∗ was observed by different investigators [110,112]. Overall, AML with inv(16) showed a miRNA signature that sometimes overlapped with t(8;21) AML; this is not surprising as both these AML subtypes belong to the CBF group [111].

In AML with *FLT3*-internal tandem duplication (*FLT3-ITD*), miR-155, miR-10a, and miR-10b were found to be upregulated [111,114].

In AML with *NPM1* mutations, a specific miRNA-based expression signature was revealed with upregulation of miR-10a and b, members of the let-7 and miR-29 families, miR-15a/16-1 and miR-17-18a-19a-20a clusters, and downregulation of miR-204 and miR-128a, predicted to target *HOX* genes known to be upregulated in *NPM1* mutated AML [114,115]. A further study was based on an integrative approach based on both microRNA and gene expression profiles. Several interesting microRNA-target mRNA interactions, such as IRF2-miR-20a, KIT-miR-20a, and MN1-miR-15a, were identified. This study also showed a deregulated expression of tumor suppressor microRNAs, such as miR-29a and miR-30c, that seem to be involved in sensitivity to therapy [116].

In AML with balanced 11q23 translocations and *KMT2A* (*MLL1*) rearrangements, the downregulation of several tumor suppressor miRNAs such as miR-34b, miR-15a, the let-7 family, and miR-196, targeting several known target genes such as *CDK4* and *CCNE2*, *BCL2*, *RAS*, and *HOX* genes was reported; other evidence showed that AML with the *MLL* rearrangement were characterized by the loss of miR-10a, miR-331, and miR-340 expression [110]. Other authors revealed the overexpression of miRNAs from polycistronic cluster miR-17-92, and a minimal class predictor with only seven miRNAs (miR-126, -126∗, -224, -368, -382, 17-5p, and -20a) was identified [112]. Leukemic cells with higher expression levels of miR-17-92 showed arrested differentiation and increased proliferation in concomitance with reduced expression of p21, a downstream target of polycistronic miR-17-92 [117].

In AML with *IDH2* mutations, a specific signature for R172 *IDH2*-AML was identified with the upregulation of miR-125b, which targets the *TP53* gene and inhibits myeloid differentiation, miR-1 and miR-133, involved in embryonic stem-cell differentiation, and downregulation of miR-194-1, miR-526, miR-520a-3p, and miR-548b, not previously associated with normal hematopoiesis or AML [118].

In AML with *RUNX1* mutations, miR-223 and two members of the let-7 tumor suppressor family were downregulated, whereas three miRNAs with an unknown role in leukemogenesis, miR-211, miR-220, and miR-595, were found to be upregulated [119].

MiRNA expression analysis performed on normal karyotype AML (CN-AML) revealed a prognostic relevant miRNA signature, as the upregulation of miR-181a/b and miR-124, miR-128-1, miR-194, miR-219-5p, miR-220a, and miR-320 was associated with a low or increased risk of failure to achieve complete remission (CR), of relapse or death, respectively [120]. Some miR-181 putative targets were genes involved in innate immunity, encoding interleukins, caspases, and Toll-like receptors [120].

### 6.2. Acute Lymphoblastic Leukemia

miRNAs deregulation is a common event in B- and T-cell malignancies in which they act as either oncomiRs or tumor suppressors [94,122,131]. A different miRNA expression profile, mostly based on miR-92a, miR-100, miR-125a-5p, miR-128a, miR-181b, miR-196b, and let-7e was revealed when comparing B-ALL lymphoblasts to normal CD34+ cells [132]. In ALL, the most frequently altered miRNAs are miR-181 cluster that is reported as upregulated by several studies and is considered a crucial oncomiR in childhood ALL [122]; miR-155, that induces pre-B cells clonal expansion and is overexpressed in different pediatric ALL subtypes [107]; miR-128b, that allows differentiation with AML cases and is downregulated in ALL with the *MLL-AF4* translocation [105,121]. In *MLL*-rearranged ALL, miR-708 and let-7b downregulation are also frequently detected, probably because of DNA hypermethylation caused by the MLL fusion protein itself. Other frequently deregulated miRNAs in ALL are miR-100, miR-125b, miR-99a, miR-126, and let-7c that are overexpressed in *ETV6-RUNX1* patients [122,123], whereas miR-181a was found as markedly downregulated in this subtype of ALL [124,125] (Table 1). In *BCR-ABL1* positive ALL cases, miR-125b expression level is downregulated at diagnosis, but significantly overexpressed after about a month. Moreover, miR-203 is silenced through epigenetic mechanisms [126]; it has been shown that by enhancing miR-203 expression, *BCR-ABL1* transcript level is reduced, cell proliferation is inhibited, and resistance to TKI can be overcome [133]. Regarding ALL with hyperdiploid karyotype, miR-222, miR-223, miR-374, miR-660, miR-98, and miR-511 were found upregulated, probably as a consequence of their mapping location in chromosomes X and 10 that are frequently present as extra copies [122].

Moreover, miRNAs expression profile data allowed the discrimination between T and B-lineage ALL based on several miRNAs such as miR-148, miR-151, and miR-424 [134]. In T-ALL, the oncogenic miR-17-92 cluster was found to be overexpressed as a consequence of the t(13;14)(q32;q11) translocation, which juxtaposed the miR-17-92 locus next to the enhancer of the T-cell receptor alpha/delta locus [135] (Table 1). miR-708 was found upregulated when comparing T-ALL with healthy individuals and downregulated when comparing T-ALL with different leukemia subtypes [122]; miR-708 downregulation is a poor prognostic factor of T-ALL, as it induces an increased expression of CD47 and promotes the evasion of leukemic cells from macrophage-mediated phagocytosis [136]. Another miRNA frequently dysregulated in T-ALL is miR-196b that is probably upregulated as a consequence of its mapping position between *HOXA9* and *HOXA10* genes that are often overexpressed [137]. Other miRNAs found to be dysregulated in T-ALL were miR-128, the oncogenic cluster miR-181, and the tumor-suppressive miR-29 [128,129,130]. Moreover, in childhood, ALL three main miRNAs were frequently reported as prognostic markers: miR-150, miR-99a, and miR-708. Low levels of miR-150 expression were associated with poor prognosis as correlating with relapse, high-risk and high WBCs at diagnosis; the association of miR-150 downregulation and poor prognosis was also revealed in other hematologic malignancies as AML, CLL, and different lymphoma subtypes [94]. Regarding miR-99, different studies revealed that upregulation and downregulation were correlated with favorable and poor prognosis, respectively [138,139]. Finally, miR-708 upregulation was associated with a good prognosis, as lower relapse risk, low WBC count, and better overall survival were detected in ALL cases; on the contrary, miR-708 downregulation was revealed in poor prognosis subtypes as T-ALL and cases with *MLL* gene rearrangement [122,139]. Finally, in ALL with *TCF3-PBX1* fusion, the deregulation of several miRNAs was also revealed (Table 1) [123,127].

### 6.3. Chronic Myeloid Leukemia

Regarding the involvement of miRNAs in chronic myeloid leukemia (CML) pathogenesis, recently it has been shown that miR-155 was highly up-regulated in CD34^+^ CML cells and allowed to evade growth-inhibitory effects of the TGF-β1 and bone morphogenetic protein (BMP) signaling; these findings provided new perspectives for miR-155 as a potential target for CML therapy [140]. Moreover, recent data revealed that miR-300 is a tumor suppressor miRNA inducing quiescence in CML leukemic stem cells (LSCs) [141], and that miR-126-3p influences both quiescence and self-renewal of CML LSCs [142] (Table 2). Another recent study showed a global decrease in microRNA levels in LSC-enriched CD34^+^CD38^−^CD26^+^ and HSC from CML-CP patients compared to those from healthy donors HSC [143]. Previous findings showed that microRNAs have also been implicated in CML progression, response to treatment, or TKI resistance [144,145,146,147,148,149,150]. A study by Edurne San José-Enériz et al. identified a group of 19 miRNAs that may predict clinical resistance to IM in patients with newly diagnosed CML [151]. Another study revealed that miR-30 induces the degradation of BCR/ABL1 mRNAs by binding directly to their 3′UTR, which was downregulated in CML patients less responsive to IM [152]. Lower expression levels of several different miRNAs such as miR-26a, miR-29c, miR-130b, miR-146a, miR-142-5p, and miR-365a-3p were identified in the peripheral blood or BM samples of CML patients who failed to respond to TKI treatment [153,154,155]. Another miRNA, miR-153-3p, was identified as downregulated in IM-resistant CML cells, and its upregulation significantly increased drug sensitivity and decreased the IM-resistant CML cells’ survival [156]. The downregulation of miR-153-3p, which directly targets B-cell lymphoma-2-mediated (Bcl-2), reduces sensitivity to IM and attenuate IM-induced apoptosis in CML. These data showed that the employment of miR-153-3p-mimic transfection combined with IM therapy might represent a promising strategy for patients with low TKI sensitivity [156]. A global transcriptome profile analysis performed on CML stem cells at diagnosis identified miR-185 as one of the most deregulated miRNAs, with a significant reduction in TKI non-responders compared with responders. The miR-185 restored expression impaired survival of TKI resistant cells, therefore miRNA targeting in combination with conventional TKI therapy may represent an efficient strategy for overcoming drug resistance in CML [156].

In contrast, another recent investigation did not find any significant differences in miRNA expression patterns between TKI responder and non-responder patients [162]. Different studies analyzing miRNA expression profiles at CML diagnosis and in cases showing resistance considered only a small series of CML cases and produced contrasting results. More extensive analyses are therefore needed to verify whether the aforementioned miRNAs may be used to discriminate between the responders and non-responders among CML patients as well as the predictive biomarkers for TKI resistance. The miRNA involvement has also been investigated in CML to identify possible biomarkers for TKI discontinuation; two miRNAs, miR-148b and miR-215, showed downregulated expression in CML cases with successful IM discontinuation, suggesting that these miRNAs may contribute to immune surveillance in CML patients showing safe TKI discontinuation [157,163].

### 6.4. Chronic Lymphocytic Leukemia

MiRNA transcription alterations have been shown in the tumor microenvironment of B-cell malignancies. miRNAs are involved in the regulation of B lymphocyte development and can be altered in different B-cell malignancies. Several studies showed that miRNAs act on various targets playing critical roles in the CLL pathogenesis, such as *BCL2*, *C-FOS*, *C-MYC*, *TP53*, *TCL1*, and *STAT3*. Both intracellular and exosomes miRNAs induce the B cells and B cell antigen receptor (BCR) activation, stimulate CLL cell progression, and could therefore be used as potential diagnostic and therapeutic biomarkers for CLL. To date, several differentially expressed miRNAs have been identified in different studies on CLL based on miRNAs transcriptional profiling (Table 2). In the pilot study by Calin et al., a 13 miRNA signature was identified in CLL patients with high ZAP70 expression and the unmutated status of the variable region of the immunoglobulin heavy chain (*IGHV*) [158]. Moreover, other studies defined specific miRNA signatures in CLL with karyotype alterations such as del(13q), trisomy 12, del(17p), and del(11q) [159,161] (Table 2). In about two-thirds of CLL cases, B-cell proliferation is stimulated by miR-15a and miR-16-1 downregulation as a consequence of 13q14 deletion; an inverse correlation between miR-15a/16-1 and the antiapoptotic gene *BCL2* expression has been observed, while CLL cell lines with miR-15a/16-1 downregulation showed an increased *BCL2* expression and resistance to apoptosis. Furthermore, low miR-15a-5p and miR-16-5p levels induce the upregulation of another target, *TP53*, that activates the expression of miR-34b-3p and miR-34c-5p and causes the reduction of ZAP-70 levels, leading to an indolent B-CLL phenotype [160]. Several miRNAs such as miR-181, miR-30d, and let-7a were differentially expressed between CLL lymphocytes and CD19+ normal cells [164], whereas another study revealed a reduced expression of miR-125b in both aggressive and indolent CLL [165]. Other miRNAs frequently involved in CLL and other B-cell malignancies are miR-150, miR-155, and the miR-17-92 cluster; they regulate the expression of crucial transcription factors involved in normal or malignant B-cells development [50,166,167]. miR-150 is considered a lymphopoietic-specific miRNA, as its overexpression inhibits the pro-B to pre-B transition, probably by targeting forkhead box P1 (*FOXP1*) and GRB2-associated binding protein 1 (*GAB1*), an important transcription factor involved in B-cell differentiation [168,169,170]. miR-155 was found to be overexpressed in both cells and plasma from CLL cases and resulted to be associated with poor prognosis and disease progression [50]. Therefore, both miR-155 and miR-150 interfere with B-cells differentiation and are involved in CLL pathogenesis. A recent study investigated a possible correlation between the miR-155/miR-150 network and clinical parameters in CLL patients, revealing its association with overall survival and CLL progression [171]. MiR-17/92 is another oncogenic miRNA with a crucial role in CLL pathogenesis and progression, being frequently upregulated and targeting different transcripts as the proapoptotic *BCL2L11* and the tumor suppressor *PTEN* [172]. Moreover, miR-29 was found upregulated in CLL cases compared to healthy individuals suggesting that it can act as an additional oncomiR; its expression was correlated to that of *TCL1*, a known oncogene with a crucial role in aggressive CLL cases. *TCL1* induces *AKT* activation and inhibits DNA methyltransferases Dnmt3A and Dnmt3B, reducing DNA methylation in CLL cells [173,174]. MiR-29 family deregulation has been specially revealed in exosomes from plasma of CLL patients, as mentioned above [51], and could therefore be employed as a useful biomarker for CLL diagnosis and progression. However, a recent study showed that all miR-29 family members (miR-29a, miR-29b, and miR-29c) were consistently downregulated in the CLL microenvironment as a consequence of BCR activation and seemed to correlate with a significantly shorter overall survival of CLL patients. Tumor-Necrosis Factor Receptor-Associated Factor 4 (*TRAF4*) has been identified as a novel direct target of miR-29s, and higher *TRAF4* levels result in downstream NFkB signaling activation [175]. Finally, some evidence showed that miRNAs could be employed as suitable biomarkers for assessing response to treatment in CLL patients. It has been shown that the treatment with “BCR inhibitor” ibrutinib induces the downregulation of several miRNAs involved in B cell activation, as miR-22, miR-34a, miR-146b, and miR-181b, whereas the expression of several target genes including *ARID1BATM*, *HDAC1*, *CYLD*, *FOXP1*, *IBTK*, *ARID2*, *PTEN*, and *SMAD4* is activated [176]. The therapy with ibrutinib or idelalisib also seemed to disrupt *TRAF4* activation induced by miR-29 deregulation [175].

## 7. miRNAs as Therapeutic Targets

miRNAs play a crucial role in regulating leukemic stem cells and the pathogenesis of hematological malignancies. Some evidence showed the crucial role of miRNAs as regulators of gene expression, biomarkers for diagnosis, prognosis, and progression, as well as molecules with the potential therapeutic application. Nowadays, miRNAs are considered promising therapeutic targets in leukemia because their silencing or inhibition does not interfere with normal HSCs function [177]. The modulation of oncogenic or tumor-suppressive miRNAs may, therefore, allow the development of novel therapeutic strategies. Two commonly investigated therapeutic approaches that allow easy modulation of miRNA levels are miRNA mimics and anti-miRNAs or miRNA-antagomiRs. miRNA mimics are small RNAs molecules that resemble miRNA precursors and can regulate the expression of target proteins; they are delivered to cells by synthetic vectors that avoid degradation and stimulate cellular uptake [178]. Anti-miRNAs are synthetic molecules complementary to endogenous miRNA that can interfere and inactivate target oncogenic miRNAs [179]. In AML, overexpression of the tumor suppressor miR-29b in blast cells was achieved using a nanoparticle-based delivery system and induced the inhibition of leukemic cell proliferation [180]. As for MM, administering miR-15a/16-1 to in vivo xenograft models showed a good efficacy, while the employment of miR-34 synthetic mimics induced apoptosis and the inhibition of MM cells in vitro [181]. MiR-16-5p mimics or lentiviral vectors were also employed in different CLL studies performed on mouse models to restore miR-15a-5p and miR-16-5p expression levels, showing B-cell cycle arrest, decreased cell viability, or induction of apoptosis [182,183]. Moreover, an in vitro synergetic action of miR-16-5p mimics and different chemotherapeutic agents was observed in the induction of apoptosis [182]. The use of lentiviral vectors for the in vivo restoration of miR-15a-5p and miR-16-5p seemed to produce low systemic toxicity and few off-target effects [183]. Another example of miRNA therapy in CLL is represented by miR-181a-5p and miR-181b-5p mimics that induce a significant increase in apoptosis when transfected in leukemic B cells from patients with wild type *TP53* and reduce leukemic cell expansion by inhibiting *TCL1A*, *AKT*, and phosphorylated *ERK1* and *ERK2* [184]. An example of a miRNA inhibitor currently in phase 1 clinical trial is MRG-106, a miR-155 inhibitor, now being tested in CLL, diffuse large B-cell lymphoma (DLBCL), and adult T-cell leukemia (NCT02580552). Another anti-miRNA is represented by antagomiR-17-5p that has been in vitro administrated to MEC-1 cells, significantly reducing miR-17-5p expression levels and cell proliferation and showed efficacy in tumor growth inhibition when injected in immunodeficient mice [185]. Interestingly, oncogenic miRNAs such as miR-22, miR-34a, miR-146b, and miR-181b were shown to be significantly decreased in response to therapy with ibrutinib in CLL patients. Knockdown of endogenous miR-34a and miR146b by anti-miRs strategy stimulated tumor suppressors transcription and inhibition of cell proliferation, confirming that these miRNAs target a subset of the tumor suppressor transcripts that were upregulated in response to ibrutinib [176]. As for the in vivo delivery of miRNA mimics and antagomiRs, besides lentiviral systems, antibody-based strategies have also been proposed by conjugating the selected miRNA with antibodies specific for characteristic markers of leukemic cells; these kinds of “vehicles” have been addressed to CD38 and ROR1 antigens expressed on the surface of leukemic B-cells [186,187]. A novel system based on lipid nanoparticles conjugated with an anti-CD38 monoclonal antibody has shown to be highly efficient in transferring miRNAs into leukemic cells, with miR-26a being the most effective in stimulating cell apoptosis [187]. The targeted delivery of miR-29b to ROR1-expressing CLL cells in vivo resulted in enhanced B-cells survival and reduced cell cycle deregulation [186].

However, miRNAs-based therapeutic approaches should be considered cautiously, accurately selecting the miRNAs to target, as some miRNAs can be oncogenic or tumor-suppressive in different conditions. For example, miR-125b affects tumor suppressors in many solid tumors and is an oncomiR in hematologic malignancies [188,189]. Moreover, the mechanism of miRNAs regulation and function has not yet been completely clarified; therefore, the development of miRNA-based treatment strategies should be carefully evaluated to guarantee the safe use of miRNAs therapy in the clinic. In conclusion, more efforts are required to improve miRNA-based therapy specificity and test their efficacy in combination with conventional approaches, avoiding toxicities and off-target effects.

## 8. Conclusions

Increasing evidence shows that miRNAs have a role as potential biomarkers in leukemia, allowing a better subtype classification, prognostic stratification, and predicting the response to treatment. An ideal biomarker should have a specific expression in patients compared to normal controls, should allow early diagnosis and minimal residual disease monitoring during the follow-up, with a possible non-invasive, simple and accurate detection method. About this, miRNAs can be extracted and analyzed from peripheral blood or bone marrow cells of leukemia patients, while circulating miRNAs can be examined by non-invasive methods based on liquid biopsy analysis and usually show expression profiles overlapping with neoplastic cells (Figure 1). To date, a large number of studies have identified different, but not always overlapping miRNAs as putative biomarkers in the same leukemia subtype, often producing contrasting data. Several miRNAs are frequently dysregulated in leukemia, such as the miR-29, let-7, and miR-15a/miR-16-1 clusters (Figure 2). Among the most frequently altered miRNAs, increasing studies show that miR-155 is involved in the pathogenesis of different myeloid and lymphoid leukemia and may be a useful putative biomarker for liquid biopsies analysis.

## Figures and Tables

**Table 1:** miRNAs most frequently involved in acute leukemia pathogenesis.

AML	miRNAs	Expression Data	References
t(15;17)(q24;q21) *PML-RARA*	miR-127, miR-154, miR-154∗, miR-299, miR-323, miR-368, miR-370, miR-382, miR-134, miR-376a, miR-127, miR-299–5p, miR-323, miR-224	Upregulation of miRNAs mapping in in the 14q32 imprinted region	[110,111,112]
t(8;21)(q22;q22.1) *RUNX1-RUNX1T1*	miR-126, miR-146a, miR-133a, let-7b, let-7c	Overexpression of miR-126 and miR-146a; downregulation of miR-133a let-7b and let-7c	[111,112,113]
inv(16)(p13.1q22) *CBFB-MYH11*	miR-99a, miR-100, miR-224, miR-126	miRNA signature sometimes overlapping with t(8;21) AML	[110,111,112]
*FLT3-ITD*	miR-155, miR-10a, miR-10b	Upregulation	[111,114]
Mutated *NPM1*	miR-10a and b, let-7, miR-29, miR-15a/16-1, miR-17-18a-19a-20a, miR-204 and miR-128a	Upregulation of miR-10a and b, let-7, miR-29, miR-15a/16-1, and miR-17-18a-19a-20a; downregulation of miR-204 and miR-128a	[114,115,116]
*MLL* rearranged	miR-34b, miR-15a, let-7 family, miR-196, miR-10a, miR-331, and miR-340, miR-17-92, miR-126, -126∗, -224, -368, -382, 17-5p, and -20a	Downregulation of miR-34b, miR-15a, let-7, and miR-196; upregulation of miR-17-92, miR-126, -126∗, -224, -368, -382, 17-5p, and -20a	[110,112,117]
Mutated *IDH2*	miR-125b, miR-1, miR-133, miR-194-1, miR-526, miR-520a-3p, miR-548b	Upregulation of miR-125b, miR-1 and miR-133; downregulation of miR-194-1, miR-526, miR-520a-3p, and miR-548b	[118]
Mutated *RUNX1*	miR-223, let-7, miR-211, miR-220, miR-595	Downregulation of miR-223 and let-7; upregulation of miR-211, miR-220, and miR-595	[119]
Normal karyotype	miR-181a/b, miR-124, miR-128-1, miR-194, miR-219-5p, miR-220a, miR-320	Upregulation	[120]
**ALL**	**miRNAs**	**Expression Data**	**References**
*MLL* rearranged	miR-128b, miR-708, let-7b	Downregulation	[105,121]
t(12;21)(p13;q22) *ETV6-RUNX1*	miR-100, miR-125b, miR-99a, miR-126, let-7c, miR-181a	Upregulation of miR-100, miR-125b, miR-99a, miR-126, let-7c; downregulation of miR-181a	[122,123,124,125]
t(9;22)(q34;q11) *BCR-ABL1*	miR-125b-2, miR-203	Overexpression of miR-125b-2; downregulation of miR-203	[126]
hyperdiploid karyotype	miR-222, miR-223, miR-374, miR-660, miR-98 and miR-511	Upregulation	[122]
t(1;19)(q23;p13) *TCF3-PBX1*	miR-126, miR-146a, miR-511, miR-545, miR-365, miR-24, miR-30d, miR193, miR-181, miR-708	Downregulation of miR-126, miR-146a, miR-511, miR-545, miR-365, miR-24, miR-30d, miR193; upregulation of miR-181, miR-708	[123,127]
T-ALL	miR-17-92, miR-708, miR-196b, miR-128, miR-181, miR-29, miR-150, miR-99a and miR-708	Overexpression of miR-17-92, miR-708, miR-196b, miR-128, miR-181; Downregulation of miR-29	[128,129,130]

**Table 2:** miRNAs most frequently involved in chronic leukemia pathogenesis.

CML	miRNAs	Expression Data	References
pathogenesis	miR-155, miR-300, miR-126-3p	Upregulation of miR-155, miR-300; Downregulation of miR-126-3p	[140,141,142]
TKI resistance	miR-30, miR-26a, miR-29c, miR-130b, miR-146a, miR-142-5, miR-365a-3p, miR-153-3p, miR-185	Downregulation	[152,153,154,156]
TKI discontinuation	miR-148b and miR-215	Downregulation	[152,153,154,156,157]
**CLL**	**miRNAs**	**Expression Data**	**References**
13q deletion	miR-15a/miR-16-1 cluster, miR-34a/miR-34b/miR-34c cluster	Downregulation of miR-15 and miR-16; upregulation of miR-34 cluster	[98,158,159,160]
Trisomy 12	miR-181a	Upregulation	[159]
17p deletion	miR-15a, miR-21, miR-34a, miR-155, and miR-181b	Upregulation of miR-21, miR-34a, miR-155, miR-181b	[161]
11q deletion	miR-34b/miR-34c cluster	Downregulation	[160]
